# A Scalable Device for Undisturbed Measurement of Water and *CO*_2_ Fluxes through Natural Surfaces

**DOI:** 10.3390/s23052647

**Published:** 2023-02-28

**Authors:** Giuliano Vitali, Marco Arru, Eugenio Magnanini

**Affiliations:** Department of Agricultural and Food Sciences, Alma Mater Studiorum-University of Bologna, 40127 Bologna, Italy

**Keywords:** *CO*_2_, evapo transpiration, surface respiration, low-power Internet of Things

## Abstract

In a climate change scenario and under a growing interest in Precision Agriculture, it is more and more important to map and record seasonal trends of the respiration of cropland and natural surfaces. Ground-level sensors to be placed in the field or integrated into autonomous vehicles are of growing interest. In this scope, a low-power IoT-compliant device for measurement of multiple surface $CO_{2}$ and WV concentrations have been designed and developed. The device is described and tested under controlled and field conditions, showing ready and easy access to collected values typical of a cloud-computing-based approach. The device proved to be usable in indoor and open-air environments for a long time, and the sensors were arranged in multiple configurations to evaluate simultaneous concentrations and flows, while the low-cost, low-power (LP IoT-compliant) design is achieved by a specific design of the printed circuit board and a firmware code fitting the characteristics of the controller.

## 1. Introduction

Climate change in a few years is moving from a mere opinion to a scary scenario. Projects oriented to the proposal of solutions for the mitigation of greenhouse gas (GHG) emissions have increased considerably while ruling tools and constraints are being adopted by a growing number of countries [1]. Most of such efforts are based on indicators and estimates of point and non-point emissions from human activities and net fluxes of natural surfaces and crops, which need to be refined and monitored [2]. Surface fluxes have been measured for years in a number of different ways, both directly and indirectly, and are mostly focused on more active terrestrial systems, where water, the primary resource for life, bolsters vegetation and allows the practice of agriculture. GHG balance in vegetated areas (both natural and cropped surfaces) is the result of dynamics of of intertwined processes involving living organisms and bio-geochemical components. While carbon dioxide ($CO_{2}$) emissions characterise oxidative processes, methane ($CH_{4}$) characterises reductive conditions, occurring in oxygen-poor and water-rich environments. This is the main reason why most of the studies focus on $CO_{2}$ (despite the high GHG power of methane) at middle latitudes. Another important GHG gas is given by water vapour (WV), which is often undervalued probably because of its ubiquity. Nonetheless, water and $CO_{2}$ are closely bound to one another—they take part in the metabolism of every temperate ecosystem. $CO_{2}$ flow at the root level dissolved in soil water has been known for several decades [3] and at the base of soil natural fertility and resilience. $CO_{2}$ soil content and outflow from soil surface are related to root and micro-organism activity, which depend on temperature, water content and soil aeration [4], which in turn are related to soil texture and structure (e.g., aeration).

### 1.1. $CO_{2}$ Flux

$CO_{2}$ budget is given by net assimilation rate resulting from incomes, mainly driven by photosynthesis (biomass assimilation), and outcomes (oxidation), largely ascribed to consumers, detritivores and decomposers (animals, fungi), and usually referred to as ‘respiration’. The carbon content of a given surface (including hypogean and epigean layers) is both the state of the system and the result of a long-lasting accumulation process. $CO_{2}$ flux measurement has been a relevant subject of research for years, with often radically different approaches on the different surfaces, especially forests and soil, which were recently a focus for understanding the possibility of carbon sequestration. $CO_{2}$ concentration is commonly observed with IR sensors (e.g., IRGA—Infrared Gas Analyzer), which can be used to observe the flow at the scale of a single leaf blade, while the most popular ones consist of the use of chambers enclosing a whole plant or on capping shallow vegetation surfaces such as lawns and crops [5,6]. Despite their different designs (e.g., [7]),they disturb the measurement environment [8,9] and need corrections in order to be introduced [10,11].

### 1.2. WV Flux

WV outflow from vegetated surfaces is commonly known as EvapoTranspiration (ET), a lumped concept used in forest and crop modelling to compute water balance. ET is mostly estimated from atmospheric data using empirical formulas; the physically based equation of Penman and Monteith is the sole factor allowing a reliable estimate of hourly data on the basis of air temperature, relative humidity, wind velocity at 2 m height and solar radiation, but it still refers to potential ET. The estimate of effective ET relies on the far more complex parameterisation of canopy water status and structure. A direct method to measure (effective) ET is based on lysimeters, allowing one to evaluate the loss of weight (of water) of a crop plot or single plant (such as a pot plant). Lysimeters are among those devices that could also estimate WV inflow, determined by condensation on the surfaces, e.g., due to radiative cooling of surfaces at nighttime in clear sky conditions [12].

### 1.3. Combined Flux Estimates

$CO_{2}$ and ET fluxes, often studied separately, have been measured and estimated at different scales. In a larger system, the (Carbon) Net Ecosystem Exchange (NEE) and transfer through the Boundary Layer are estimated by the eddy-covariance method, involving the use of expensive point-wise tower-based systems, characterised by large spatial fluctuations [13] The system is also used to measure ET on forest canopies; however, the apparatus is costly and the data are not easy to be analysed. On the other hand, remote sensing does not seem to help yet in supplying information continuously and with an adequate resolution. In cropland, the component of water loss by transpiration has been related to biomass fixation, as most of the flow is recognised to happen through stomata, regulating simultaneously the diffusion of both gases [14]. Interest in such evaluations also grew in tree crops and vineyards to optimise yields [15] and Water Use Efficiency [16]. Simultaneous observation of $CO_{2}$ and water fluxes from vegetation, sometimes together with other gases, is of increasing interest (e.g., [15]. Both fluxes have also been estimated on large surfaces with the help of satellite sensing, calibrated by the abovementioned methods for each different surface/canopy.

### 1.4. Sensors and IoT

The progress in semi-conductor development that occurred in the last decades has increased the number of sensing technologies [17] and allowed the technology used in the lab and/or high-cost portable detectors to become available (as Commercial Off-the Shelf) for integration into low-cost devices [18]. The technology on which several studies are focusing for a while for $CO_{2}$ observation is the one based on non-dispersive infrared (NDIR) sensors [19]. Today, cheap and reliable sensors are available from several makers, and their diffusion is going to be harnessed by the Internet of Things (IoT) [20]. IoT is a technology based on two components: devices connected to the internet and a cloud computing system that allows perceiving devices as things. IoT decreases the costs of devices, allows fast prototyping and shortens the engineering process [21]. In a few years, IoT promises to transform our life, disseminating over the globe a number of devices hosting intelligent sensors (meters) and actuators. IoT is also introducing a new way of doing research, where cheap devices, probably unable to reach the accuracy of their costly cousins, may reduce time intervals and increase the spatial resolution of the observations. Therefore, such cloud-connected low-cost devices offer a brand new option for the observation and characterisation of environmental variables [22]. Recently, a rising interest has been oriented to wireless low-power technology [23], focused on the development of long-lasting devices. Such devices, inactive for most of the time, are then awakened by endogenous or exogenous events; perform the actions they were designed for, including communication; and then go back to sleep. In stand-by, such devices could require just a few uA to maintain the basic functions, such as a Real-Time Clock (RTC) or a low-consumption radio receiver operating like an alarm clock. Adopting such a simple scheme, together with a reduced duty cycle measurement (working time to sleep-time ratio), a common rechargeable battery kept charged by a small photovoltaic panel can last for years.

### 1.5. Aims and Plan

The aim of this study is to design, describe and show the performances of a scalable low-power IoT device, named ETR, to estimate the combined flux of $CO_{2}$ and WV concentrations and flux on a vegetated surface in undisturbed conditions. In this analysis, a description of the architecture of the ETR is given in Section 2, while preliminary tests are reported and discussed in Section 3; finally, we discuss the strengths and weaknesses of the solution, and draw the final conclusions.

## 2. Design of the ETR

The system design started from the following specifications:

- The system should measure $CO_{2}$ and WV concentrations in a number of points with a variable distance, ranging from a few centimetres to several metres;
- Be simple to set inside and outdoor, both on ground and on poles;
- Easy to see real-time data, time records and retrieve data;
- Durable, requiring low maintenance;
- Autonomously powered;
- Low-cost;
- With negligible environmental pollution (including radio-frequencies).

**Figure 1:**
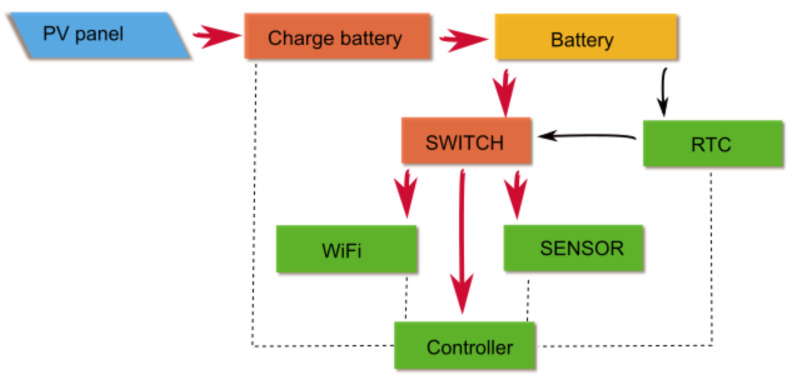
Block diagram of the device, where power lines are identified by red arrows, while dashed lines represent digital data and controls.

The block diagram of the system can be sketched as shown in Figure 1 which includes the following:

- A photovoltaic panel (8 × 12 cm, 6 V)
- A battery charging system for Li-Ion batteries;
- One Li-Ion battery (3.6 V), which constantly feeds only the RTC circuit (very-low power consuming), with the role to switch the power to the rest of the electronics;
- A switching circuit powering the remainder of the system, including the following:
- A controller;
- A sensor set;
- A WiFi interface.

A battery charge gauge, an LTC2942, was used to read the state of the battery, which is fundamental to monitor the device’s working ability and the possible needs for maintenance.

### 2.1. The Controller

The controller selected was an ESP32 (ESP32−WROOM−3ver.32D [24]), belonging to a popular family of controllers used in the development of low-power solutions for the novel Internet of Things (LP−IoT). The ESP32 was designed to achieve high power and RF performance. It is based on an Xtensa LX6 microprocessor, a 448 Kb ROM and 520 kB of RAM memory, allowing it to easily process images. It has an internal 8 Mhz oscillator with calibration, an integrated RTC timer, with an inner 16 KB SRAM and watchdog. It has an integrated 2.4 Ghz WiFi and Bluetooth and RTC integrated—in deep-sleep mode, the RTC is the only powered device. ESP32 comes with 34 programmable General Purpose I/O (GPIO) ports, a 12 bit analog-to-digital converter (ADC) and 2 digital-to-analog converters (DAC), 10 touch sensors and various interfaces (4 SPI, 2 I2C, 2I2S, 3 UART, ETH) together with encoders for Motor PWM and LED PWM.

### 2.2. The Sensor

**Figure 2:**
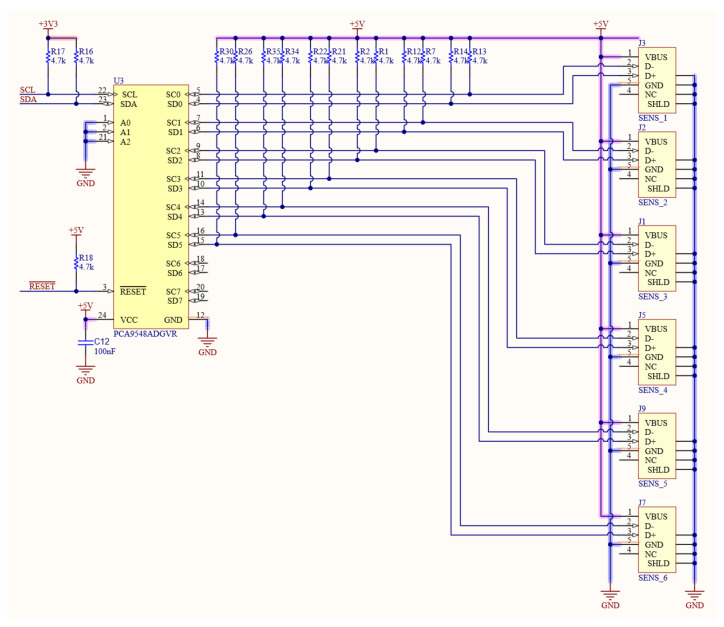
Sketch of connections of multiplexer used to cycle over sensors.

The sensor used was SCD30, a small (35 × 23 × 7 mm) low-cost sensor from Sensirion [25], hosting a Non-dispersive Infrared (NDIR) sensor for $CO_{2}$ (range: 0: 10,000 ppm, accuracy ±30 ppm +3%), with integrated measurement of relative humidity RH (range: 0:100%, accuracy ±2%), and of temperature TC (range: −40: 70 °C, accuracy ±0.3 °C), operated by a unique sensor interface SHT30 [26] with an electric capacitance linearly dependent on absolute humidity (Dw). The SCD30 comes with 6 active pins, 2 for powering (3.3–5 V, c.ca 70 mA while measuring), 3 for data (CLK, DATA, data−ready), to be used with $I^{2}C$ or Modbus protocols (selected by a dedicated pin). Further details can be found from Sensirion [25]. As in the present version of SCD30, the $I^{2}C$ address cannot be set, a $I^{2}C$ multiplexer (TCA9548A) was used (see Figure 2) to select the sensor in use. The system was designed to host 6 sensors, though the number is only bounded by the multiplexing circuit (TCA9548A itself has 8 ports).

### 2.3. The Assembly

**Figure 3:**
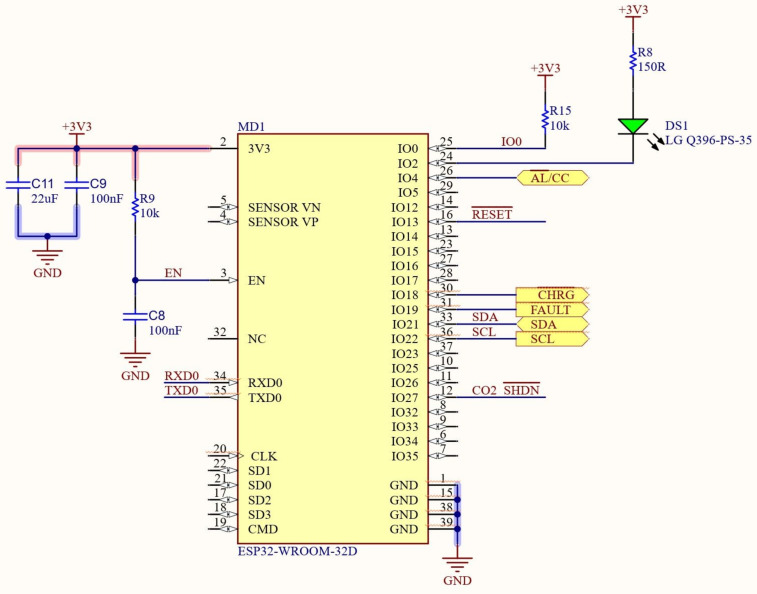
Sketch of the connections of controller-ESP32.

A specific printed circuit board (PCB) was developed to wire together the battery charger, the controller and the multiplexer. The connections to the ESP32 are shown in Figure 3.

**Figure 4:**
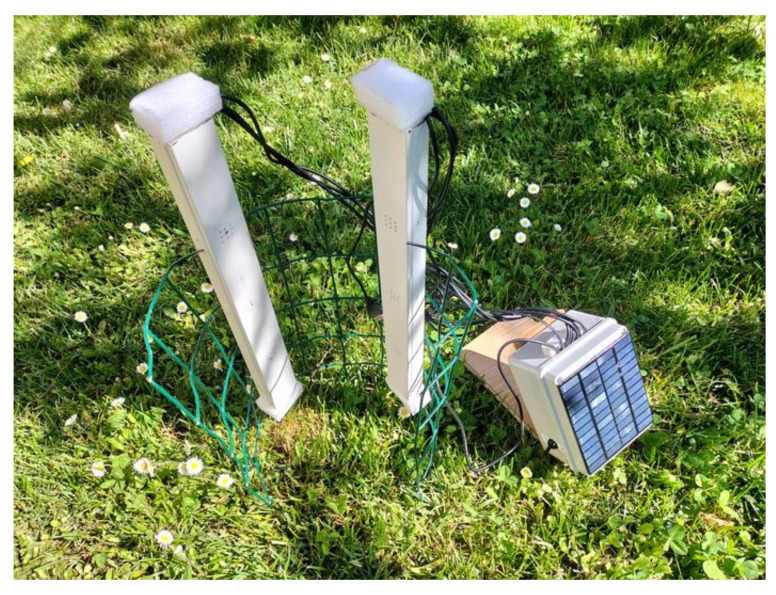
ETR device.

A specific printed circuit board (PCB) was developed to connect each sensor with a USB connector: this allows the use USB cables of different lengths (commercial USB cables reach 20 m). Commercial enclosures were used to house the boards and the sensors. The 6 sensors were set in 2 bars, at 10 cm distance. They were mounted following the directions of the producer [26]. The full ETR device is shown in Figure 4.

### 2.4. The Logic

**Figure 5:**
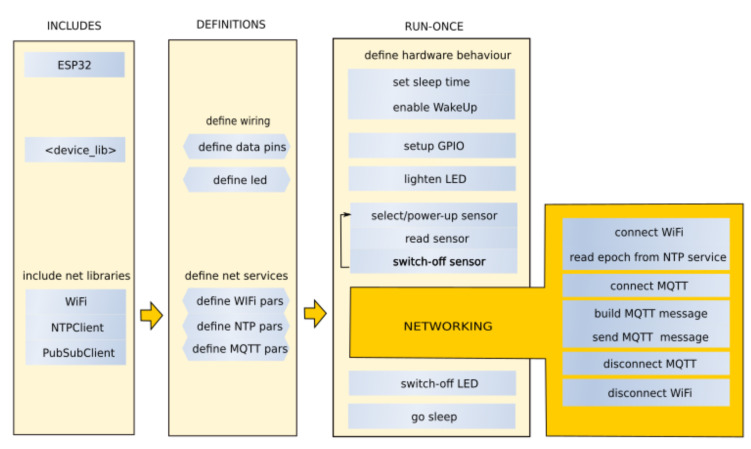
Flowchart of the code running on the ESP32 controller.

The device was designed to be used in two different ways, continuous recording or long-time records; therefore, two different versions of the firmware were developed but with a common architecture. The code was developed in C-language (by Arduino IDE [27], code available to authors upon request). Figure 5 reports the flowchart of the code used for long-time measurement. In this case, the system awakens periodically, makes the measurement and goes back to sleeping mode—this way, battery consumption is minimised. On the other hand, in the continuous recording mode, it is expected that the user manually switches on and off the apparatus, which keeps recording data, until the user does not intervene. This first mode is for autonomously powered long-time monitoring, while the second is used to monitor continuously the behaviour of a system under the control of an operator—a typical scenario is one of a field respiration chamber, which is expected to work for a short period after its closing: with this apparatus and operating mode, up to 6 chambers can be observed simultaneously. Of course, the apparatus can be used indoors, powered with USB for an indefinite time. In Figure 5, a three-column flowchart is shown. The leftmost column reports the INCLUDE section listing the libraries used, the Wire for the use of $I_{2}C$ data communication protocol [28], LTC2942 [29] to monitor battery charge, WiFi to connect to the WLAN [30], NTPclient to retrieve the timestamp (epoch) from an NTP service (based on WiFi’s UDP protocol [31]), PubSubClient to support the MQTT protocol [32] and the SCD30 library [33] to manage the sensor interface. The middle column reports the DEFINITION section, including the setting of General Purpose Input Output (GPIO) used for (digital and analog) data communication, together with the structured variables used to access devices by means of the libraries reported above. The definition section also includes the initialisation of structured variables related to the network services used: WiFi, NTP, MQTT. In the third column (RUN−ONCE), the tasks performed at run-time are reported, including the initialisation of wake-up mode and setting of sleeping time on the RTC, and the enlightening of an LED. Successively, the devices are turned on so that the sensors start warming up, the multiplexer is initialised and the reading cycle activated. Once the reading cycle is completed, the device connects to Wifi, the NTP time is updated and the MQTT message is built including sensor values and sent through the internet. Finally, every peripheral is switched off and the controller is put in deep−sleep mode, waiting for the next trigger operated from the RTC. The rightmost inset details network activities including cycles for service connection and final disconnections.

### 2.5. Data Flow

**Figure 6:**
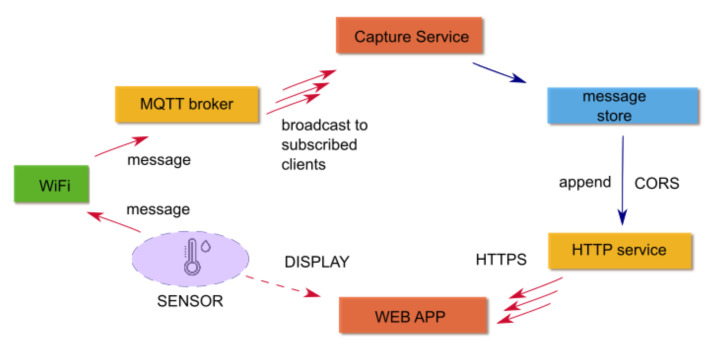
IoT communication routing used in the present application; the arrows show the true information flow, the dashed one is the one ‘apparent’ to the user.

The MQTT protocol is a lightweight bidirectional messaging system between a device and the cloud, allowing easy broadcasting of messages between things. MQTT clients are small, require minimal resources and have optimised bandwidth; so, they can be used on small micro-controllers. The MQTT protocol is a broker service. Several MQTT free services are available for free; however, they have some limits, e.g., the number of messages/topics. For this reason, a freely deployed MQTT broker, Mosquitto [34], was installed on a server. Acknowledged MQTT clients may subscribe to a TOPIC (an ASCII string where subtopics are identified by the separator “/”) and send or receive ASCII messages, which are commonly coded as JSON. The message may contain the state of a sensor/meter/device or communicate the action to an actuator. A standard structure of messages have recently been defined, e.g., by FIWARE, based on the concept of entities and Smart Data Models [35] As the MQTT service provides almost no data persistency, the task should be provided by other services provided by cloud computing [22]. In our case, to supply data storage and accessibility of historical data, a specific service based on a MQTT client was developed (not described here), which continuously collects the messages in the server. A web app (dashboard) was finally developed for the visualisation of historical and real-time data, with the latter being a mandatory feature, whenever a device comes without a built-in display. Figure 6 describes the data flow, where it is easy to see how the old-fashioned data-logger paradigm is substituted from a data stream service based on WiFi and on MQTT communications protocol (see Figure 6). The technology allows the separation between measuring and data logging processes, making data robust and fully accessible to every user.

## 3. Performances

### 3.1. A Calibration Chamber

**Figure 7:**
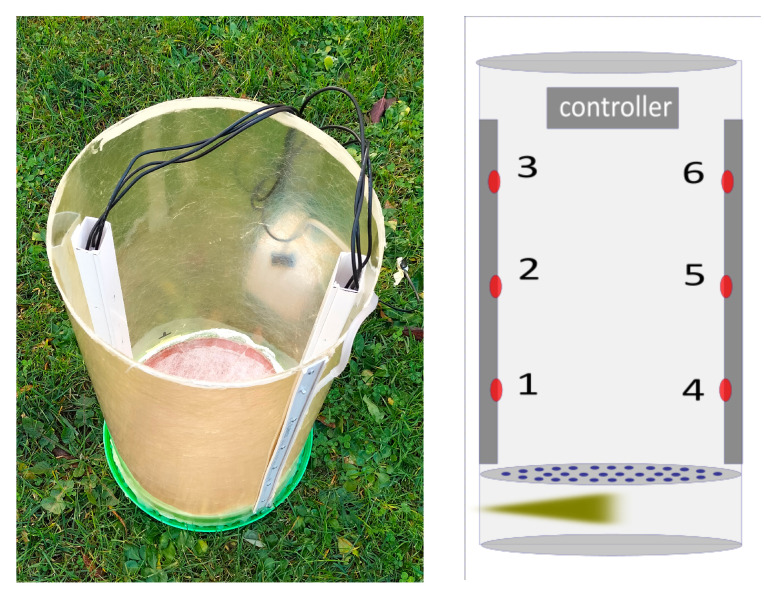
Steady-state chamber. Points 1–6 refer to the placement of the sensor.

To test the system, an open chamber was developed: it consists of a 30 cm diameter plastic cylinder, 60 cm in height, which can easily host the two sensor bars, while a cover allows it to be closed on the upper side (see Figure 7, left). The chamber was equipped with an injection point on its bottom part and separated from the upper part by a diaphragm to prevent the propagation of the turbulence of injection to the upper part of the chamber (see Figure 7, right).

**Figure 8:**
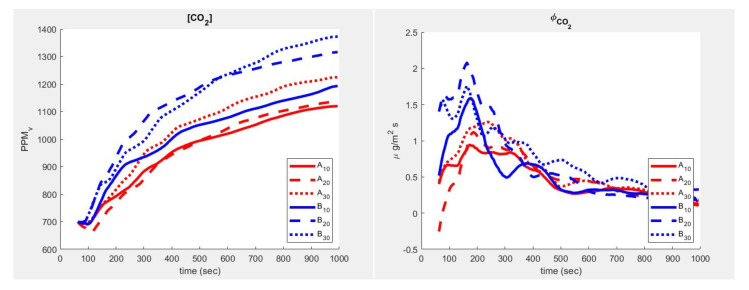
$CO_{2}$ concentration response to 1 s human breath in the open chamber.

Several tests of the sensing device were performed in the test chamber, both in the open and closed configurations, to assess the possibility of detecting differences in concentrations of $CO_{2}$ and WV. Figure 8 shows observations from a single injection. The exhaled gas, with a high concentration of $CO_{2}$ and of WV, mixes with the (room environment) air in the bottom chamber, while reducing its turbulence, then gradually diffuses to the top through the diaphragm—the chamber aims at simulating a quasi-1D diffusion—and distributes to reach a maximum concentration. The rightmost part of Figure 8 reports the fluxes computed by means of Equation (1) using each of the 2 layers (10–20 and 20–30 for each of the two bars hosting the sensors—see Figure 4). Flow (ϕ) was computed using A plain discretisation of Fick’s law and can be presented as follows:

(1) $$k_{j}\left(t\right)=\left(C_{i}\left(t\right)-C_{i}\left(t-dt\right)\right)\cdot \left(2\cdot C_{j}\left(t\right)-C_{j-1}\left(t\right)-C_{j+1}\left(t\right)\right)\cdot dz^{2}/dt$$

(2) $$\phi_{j}\left(t\right)=-k_{j}\left(t\right)\cdot \left(C_{j}\left(t\right)-C_{j-1}\left(t\right)\right)/dz$$

where *C* and ϕ are, respectively, the concentration and flow (per unit surface) of $CO_{2}$ and Dw, and *k* is the relative apparent diffusion coefficient (m^2^/s) in layer (*j*) and time (*t*).

**Figure 9:**
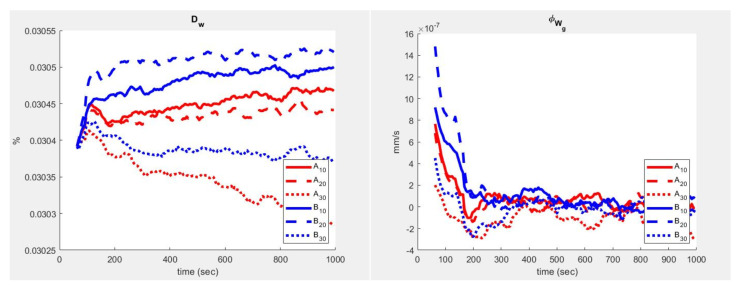
Air absolute humidity response to 1 s human breath in the open chamber.

While in the concentration curves (left side of Figure 8) the delay of the diffusive wave is almost unperceived, a more complex scenario appears in the flow estimates (Figure 8 right side). Even being outside the aims of this study to interpret the gas dynamics, it appears that when using an apparently simple device architecture (the chamber), the process already shows the complexity typical of term-fluid-dynamics of free convection. In fact, it is possible to see how a slight variation in slope in the concentration corresponds to non-negligible fluctuations in the flow (phi) of $CO_{2}$. The corresponding trend of WV is shown in Figure 9.

### 3.2. Open-Air Trials

**Figure 10:**
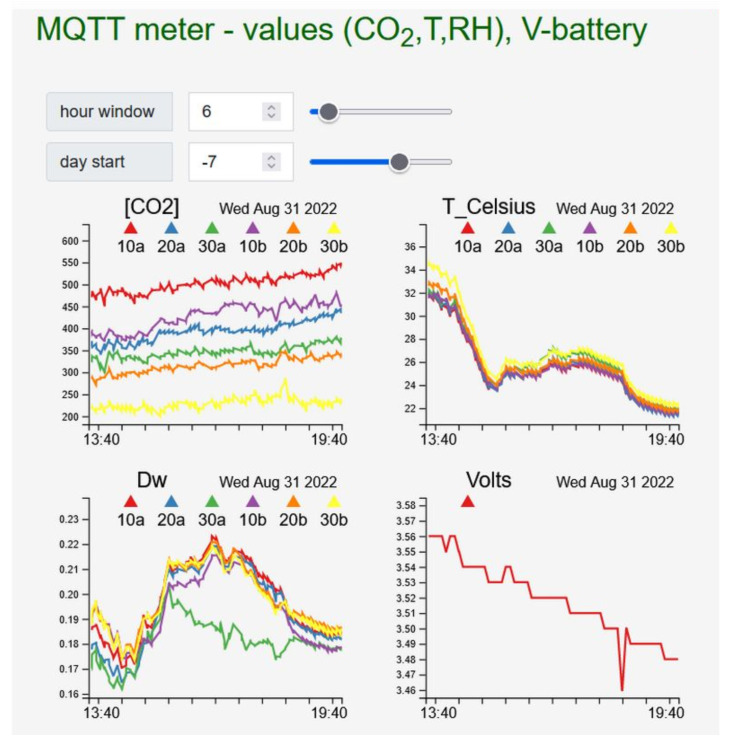
ETR device behaviour in open-air on the app GUI.

Similar measurements were made in a watered lawn: row values are shown as they appear in the dashboard of the web app in Figure 10. The graph is mainly aimed at presenting the web-app functionalities, which include the possibility to navigate through the collected data, specify the width of the time window (in hours) and to see the last point to be visualised (starting point in the GUI), giving the user the possibility to observe data several days back counting from the last visualised one. In the present version, the device makes a measurement every 20 min, after which it switches into deep-sleep mode: during this time, the photovoltaic panel recharges the battery (during daytime) for the next observation cycle.

## 4. Discussion

The ETR proved the possibility to design a cost-effective and reliable measurement device with commercial off-the-shelf components. The wireless and low-power design allows for remote monitoring of its state and sensor values, while the IoT approach allow them to be managed by cloud-computing services (e.g., [18]). The open-software choice allows setting the duty-cycle measurement to fit the purpose of observation and guarantee a long-time operation in the open-air. The modular strategy allows the ETR for easy and reliable development and up-scaling—it can easily host a number of sensors. Nonetheless, the gas sensors still suffer calibration issues, already known from literature [17]. A belief underlying the IoT approach is that using low-price sensors, though they do not guarantee low error values, they may be used to increase spatial coverage and reduce errors due to spatial variability, while data redundancy may be used for lining up data from different sensors. The need of increasing spatial density and temporal continuity of records is mandatory in environmental observations, where even long-time assessed devices still suffer non-negligible errors and data corrections [36]. Errors are particularly sneaky in experimental setups affecting the micro-climate such as closed, through-flow and open chambers [37]. The same eddy-covariance-based networks, diffused all over planet Earth, together with their historical data sets, aimed at reducing measurement errors [5,38], integrating them with satellites and modelling (including Artificial Intelligence) [2]. To the scope, portable instruments may also help in collecting ground-based information [39].

## 5. Conclusions

The evolution and the accessibility of semiconductors and of cloud technologies, allow for the advent of IoT scenario. A new family of devices can change radically the measuring concept, making it accessible also outside research institutions and company departments, making the net of environmental observation enriched by the new generation of Wireless Sensor Networks. This study focused on the development of a device for the simultaneous measurement of $CO_{2}$ and WV in points at a variable distance, investigating the possibility to estimate the flows and monitoring the values by means of a cloud-based service. The following points can be emphasised.

- The ETR can be used both indoors (by USB direct supply or regular recharge) and outdoors, where the PV cell is sufficient to keep the battery charged for a long time with an optimised duty cycle measurement; further, the system modularity allows it to be supplied by newer energy harvesting systems.
- The system can be used to arrange sensors ina variety of configurations allowing users to obtain information on flows or from a series of chambers as they are all wired to the main PCB, displaying as a limitation the distance to the I2C platform.
- The system proved to be a basis for further developments, as it can easily host more sensors, including radiation (light) sensors, for evaluating photosynthetic activities, or anemometers to estimate eddy-diffusivity-based gas flows.
- The IoT solution allows for embedding in cloud-computing the more intensive computing features, including Machine Learning.

Critical steps have been recognised in the following:

- The importance of a good PCB design is relevant for reliable exploitation of the controller and of the connected devices, especially in a low-power device development context;
- The embedded firmware is not easily portable to other controllers, as the selection of libraries and code features fitting ESP32;
- The reliability of a cloud computing system, including every service and app for data ingestion, storage and accessibility;
- The choice of sensors and of a correct experimental design;
- Unreliable external network infrastructure (e.g., WLAN)—the present device may suffer a high number of attempts to reconnect to the WLAN due to repeated failures, which could reduce the duration of the battery;
- Calibration of sensors for long-term, open-air observations is the most important point to be issued in the next development before considering the engineering of the device to increase the reliability of the surface flow estimate.

## Data Availability

Not applicable.
